# Protective efficacy of an RBD-based Middle East respiratory syndrome coronavirus (MERS-CoV) particle vaccine in llamas

**DOI:** 10.1186/s42522-022-00068-9

**Published:** 2022-06-24

**Authors:** Jordi Rodon, Anna Z. Mykytyn, Guillermo Cantero, Irina C. Albulescu, Berend-Jan Bosch, Alexander Brix, Jean-Christophe Audonnet, Albert Bensaid, Júlia Vergara-Alert, Bart L. Haagmans, Joaquim Segalés

**Affiliations:** 1 Unitat Mixta d’Investigació IRTA-UAB en Sanitat Animal, Centre de Recerca en Sanitat Animal (CReSA), Campus de la Universitat Autònoma de Barcelona (UAB), Bellaterra, Barcelona, Catalonia 08193 Spain; 2 IRTA Programa de Sanitat Animal, Centre de Recerca en Sanitat Animal (CReSA), Campus de la Universitat Autònoma de Barcelona (UAB),Bellaterra, Barcelona, Catalonia 08193 Spain; 3 Department of Viroscience, Erasmus Medical Centre, Rotterdam, CA 3000 The Netherlands; 4 Virology Division, Department of Infectious Diseases & Immunology, Faculty of Veterinary Medicine, Utrecht University, Utrecht, CL 3584 The Netherlands; 5 Boehringer Ingelheim Veterinary Research Center GmbH & Co. KG, Hanover, Germany; 6 Boehringer Ingelheim Animal Health, Global Innovation, 813 Cours du 3ème millénaire, Saint-Priest, 69380 France; 7 Departament de Sanitat i Anatomia Animals, Facultat de Veterinària, UAB, Campus de la Universitat Autònoma de Barcelona (UAB), Bellaterra, Barcelona, Catalonia 08193 Spain

**Keywords:** Animal model, Llama, Camelid, Middle East respiratory syndrome coronavirus, MERS-CoV, Multimeric protein scaffold particles (MPSP), Receptor binding domain (RBD)-based vaccine, Virus transmission, Neutralizing antibodies

## Abstract

Ongoing outbreaks of Middle East respiratory syndrome coronavirus (MERS-CoV) continue posing a global health threat. Vaccination of livestock reservoir species is a recommended strategy to prevent spread of MERS-CoV among animals and potential spillover to humans. Using a direct-contact llama challenge model that mimics naturally occurring viral transmission, we tested the efficacy of a multimeric receptor binding domain (RBD) particle-display based vaccine candidate. While MERS-CoV was transmitted to naïve animals exposed to virus-inoculated llamas, immunization induced robust virus-neutralizing antibody responses and prevented transmission in 1/3 vaccinated, in-contact animals. Our exploratory study supports further improvement of the RBD-based vaccine to prevent zoonotic spillover of MERS-CoV.

**Supplementary Information:**

The online version contains supplementary material available at 10.1186/s42522-022-00068-9.

## Main text

MERS-CoV is associated with severe pneumonia and lethal disease in humans with high case-fatality rates in the Middle East [1]. The virus still poses a public health concern since ongoing zoonotic transmission events from dromedary camels, the main source of infection, and several major travel-associated outbreaks have been documented [2].

Dromedaries are the main reservoir, although other camelid species such as llamas and alpacas are also susceptible to MERS-CoV [3–10]. Camelids, as opposed to humans, undergo a mild to subclinical infection upon MERS-CoV infection, characterized by upper respiratory tract replication and rapid clearance of the virus within 1–2 weeks after infection [11, 12]. Robust and timely innate immune responses occurring in camelids might play a crucial role in controlling MERS-CoV infection and disease development [4]. Importantly, animals showing nasal discharges and asymptomatic carriers shed abundant quantities of MERS-CoV [3, 5, 11, 12], which may result in a potential spillover to humans.

To date, commercial vaccines and therapeutics against MERS-CoV are lacking, and the World Health Organization has advised animal vaccination as a strategy to control the spread of MERS-CoV to animals and humans [13]. Different vaccine prototypes have been tested in camelids to counteract MERS-CoV, all of them focusing on the full-length or specific regions of the spike (S) protein [5, 12, 14, 15]. This protein mediates viral entry by binding to the host cell receptor dipeptidyl peptidase-4 [16] and subsequent fusion of the viral and cellular membrane. The spike protein is highly immunogenic and the main target of neutralizing antibodies and, therefore, the antigen of choice for vaccine development against MERS-CoV and other betacoronaviruses [17]. Viral-vector vaccines expressing the full-length S protein induced partial immunity and, in some instances, when exposed to MERS-CoV, reduced rhinorrhea and viral shedding in dromedaries [12, 15]. Importantly, an increase in neutralizing antibody (nAb) titers was observed after one vaccination of seropositive animals, resulting in minimum excretion of viral RNA after exposure to naturally infected camels [15]. This fact is of special relevance due to the high prevalence of seropositive camels found in the Middle East. The usage of recombinant protein vaccine candidates based on the S1 subunit have also been proposed for camelids [14]. Three administrations of an S1-based vaccine prototype conferred full protection against MERS-CoV in alpacas, as well as delayed and reduced infectious viral shedding for 3 days after intranasal challenge of dromedary camels [14]. Differences in protective efficacy between host species might be explained by the differential response to the vaccine, as evidenced by the levels of nAbs elicited [14]. Further, to mimic the natural transmission occurring in the field, we previously developed a direct-contact llama transmission challenge model to demonstrate that a recombinant S1-protein vaccine was able to block MERS-CoV transmission among camelids [5].

Here, we used the same direct-contact model to assess the efficacy of a virus-like particle vaccine to block MERS-CoV transmission in llamas. The vaccine was composed of self-assembling multimeric protein scaffold particles (MPSP) expressing the receptor-binding domain (RBD) of the MERS-CoV S protein [18]. The MPSP vaccine prototype allows the self-assembly of antigens into 60-mer particles and offers enhanced immune responses in comparison to other multivalent and monomeric recombinant vaccines [18–20]. Indeed, the proposed vaccine prototype induced strong protective immune responses that reduced MERS-CoV replication in the upper and lower respiratory tract of experimentally infected rabbits [18]. Since rabbits do not develop severe disease upon MERS-CoV inoculation as occurs in humans, nor a subclinical infection with high viral secretions that camelid reservoirs experience [21], this study provided a rationale for testing the MPSP-RBD vaccine prototype in camelids.

**Fig. 1:**
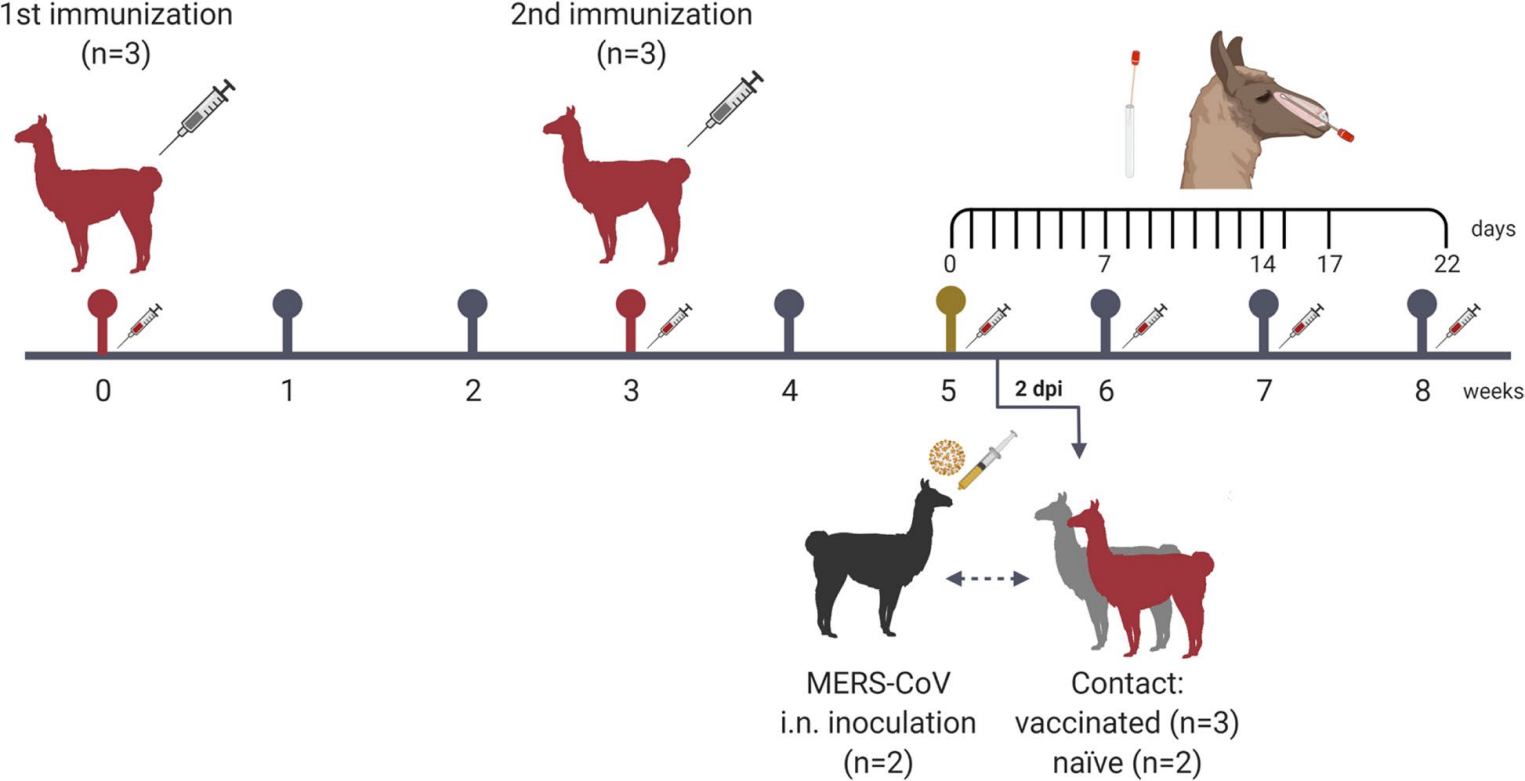
Schematic representation of the experimental design. Two llamas (black) were intranasally inoculated with MERS-CoV (Qatar15/2015) and two days later brought in contact with two naïve (grey) and three vaccinated (red) llamas. Immunization dates are shown in red timeline points and with grey syringes. MERS-CoV-inoculation procedure is stressed as a gold time point. Blood collection days are represented with a red syringe symbol on the weeks scale. Sampling scheme of nasal swabs in all animals is shown using black lines in a daily scale. Dpi, days post-inoculation; i.n., intranasal

Following a previous experimental design to test vaccine efficacy mimicking field-like conditions [5], a group of three llamas was vaccinated with two doses of the MPSP-RBD in combination with a registered adjuvant (Fig. 1, Additional Material and Methods). After prime and boost immunizations, vaccinated (*n* = 3) and adjuvant-control administrated animals (*n* = 2) were put in direct contact with naïve llamas (*n* = 2) infected with MERS-CoV (see Additional Fig. 1). Two days before mixing the groups together, naïve llamas were inoculated with MERS-CoV Qatar15/2015 strain, a clade B strain shown to replicate efficiently and be transmitted between camelids in direct contact [4, 5]. Clinical signs and body temperatures were monitored, and collection of nasal swabs for virological studies were conducted as indicated in Fig. 1 and detailed in Additional Material and Methods.

**Fig. 2:**
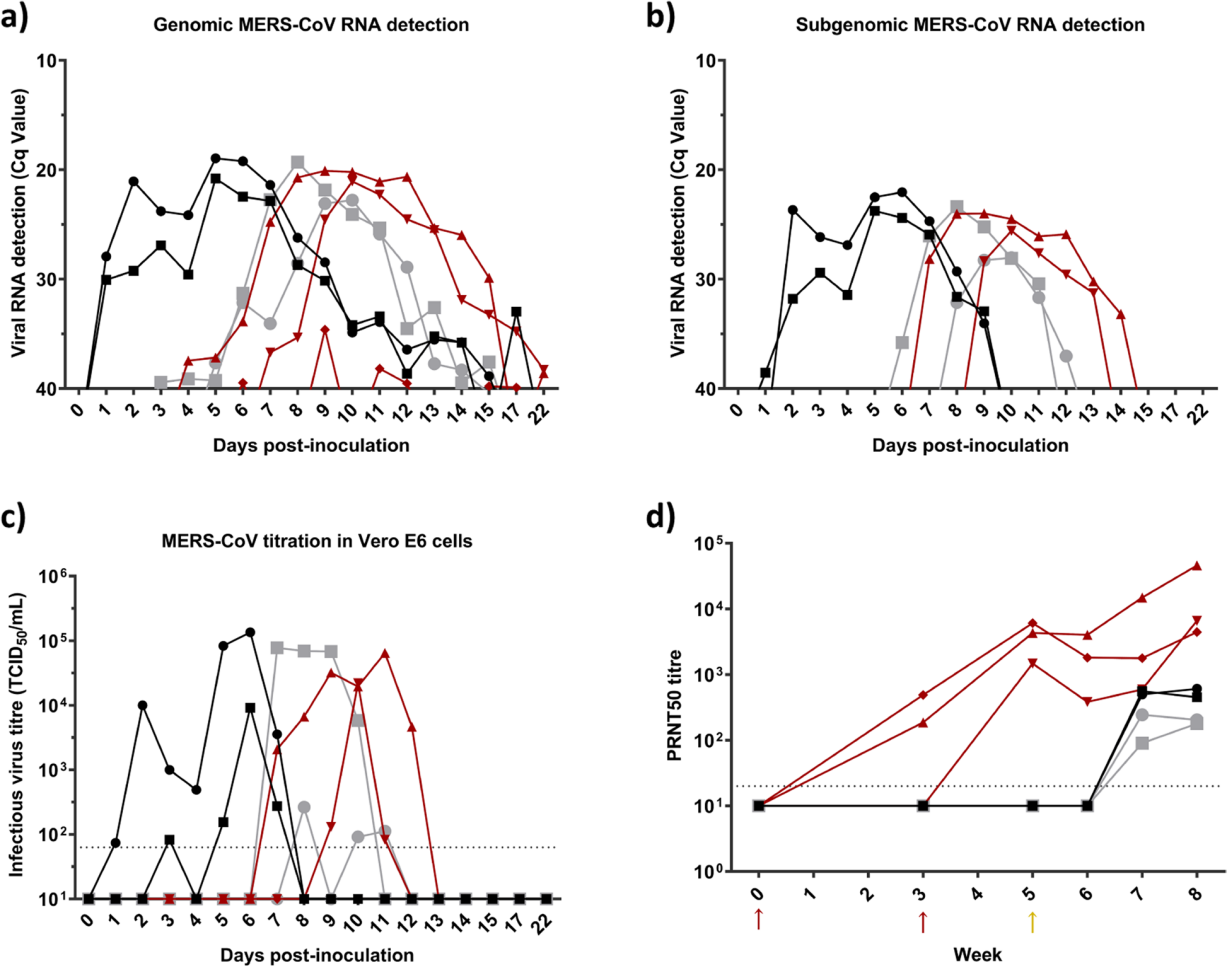
MERS-CoV RNA and infectious virus shedding and development of neutralizing antibodies in llamas. Experimentally infected llamas (black) were placed in contact with naïve (grey) and vaccinated (red) animals two days after MERS-CoV inoculation. Genomic (**a**) and subgenomic (**b**) viral RNA was quantified in nasal swab specimens collected at different times after MERS-CoV inoculation. Plot (**c**) show infectious MERS-CoV titres in nasal swabs collected on different days after MERS-CoV inoculation. Plot (**d**) displays serum neutralizing antibodies elicited against MERS-CoV in vaccinated, experimentally inoculated and in-contact naïve llamas. Each line represents an individual animal. Dashed lines depict the detection limits of the assays. Red and yellow arrows indicate the two MPSP-RBD immunizations and MERS-CoV inoculation days, respectively. Cq, quantification cycle; MERS-CoV, Middle East respiratory syndrome coronavirus; PRNT50, 50% plaque reduction neutralization titre; TCID_50_, 50% tissue culture infective dose

Rectal temperatures of all animals remained basal (37–40 °C) throughout the study (Additional Fig. 2a). None of the inoculated llamas showed clinical signs at any day post inoculation (dpi). One contact-control animal showed moderate rhinorrhea at 5–9 dpi, and one vaccinated animal from 8 to 19 dpi (Additional Fig. 2b and c, respectively). As previously reported [5], MERS-CoV-inoculated llamas had detectable genomic and subgenomic viral RNA in nasal swabs for a period of 2 weeks (Fig. 2a and b) and shed high titers of infectious virus during the first week after inoculation (Fig. 2c). These animals seroconverted for MERS-CoV and nAbs were detected from 2 weeks after infection onwards (Fig. 2d). As determined by RT-qPCR and virus titration in cell culture, MERS-CoV was transmitted to all adjuvant-administered and two out of three vaccinated, in-contact animals at 5–7 dpi (Fig. 2a, b and c). With the exception of one vaccinated llama, all animals had similar profiles in the duration and levels of viral RNA and infectious virus shedding (Fig. 2a, b and c). These results are comparable to previous ones obtained in inoculated and naïve contact animals [5]; therefore, individual differences observed in the current study may account for minor variations in viral shedding patterns of vaccinated and control-contact animals. The remaining vaccinated-contact llama was protected against MERS-CoV infection. Only minor traces of MERS-CoV genomic RNA were detected in nasal swabs of this animal along the experiment, evidencing its exposure to the virus (Fig. 2a). Moreover, subgenomic RNA was not detected at any time point of the study in this vaccinated llama and the animal did not shed infectious virus (Fig. 2b and c). Furthermore, all inoculated and in-contact naïve llamas developed a comparable neutralizing humoral response to MERS-CoV (Fig. 2d). MPSP-RBD vaccination induced high titres of virus nAbs in sera, which were boosted in 2 out of 3 animals three weeks after contact with MERS-CoV-inoculated llamas shedding high titres of infectious virus (Fig. 2d). Thus, the MPSP-RBD vaccine candidate was able to partially prevent MERS-CoV transmission among camelids, being effective in 1/3 of the animals vaccinated in this exploratory study.

Based on the enhanced immune response offered by MPSP-displayed immunogens and the in vivo protective capacity of the MPSP-RBD vaccine prototype against MERS-CoV [18], we evaluated its potential to inhibit MERS-CoV transmission among camelid reservoirs. Immunization with the MPSP-RBD formulated with a commercial adjuvant elicited nAbs to MERS-CoV but transmission was only prevented in 1/3 of the animals. Since high MERS-CoV seroprevalence and evidence of reinfection have been found in camelids [22], further studies would be needed to investigate whether MPSP-RBD administration can boost sufficient protective immune responses to MERS-CoV and decrease the transmission rate in previously exposed animals. The monomeric RBD displayed by MPSP may induce lower protective responses than a prototype shaping a trimeric conformation or the combination with other S subunits, as evidenced by the high efficacy of a previous vaccine candidate using the same adjuvant and route of administration [5]. Nonetheless, the capabilities of MPSP-RBD to prevent animal-to-animal transmission of MERS-CoV and, eventually, human spillover, seem limited.

## Supplementary Information

**Additional file 1: Fig. 1.** Animal distribution scheme inside the experimental box. Experimental groups were kept in different compartments separated by tarpaulin to prevent animal contact. Two days after inoculation procedure, the tarpaulin was removed and experimentally infected llamas (black) were then in direct contact with naïve (grey) and vaccinated (red) animals.

**Additional file 2: Fig. 2.** Temperature and rhinorrhoea after MERS-CoV exposure to llamas. MERS-CoV experimentally inoculated llamas (black) were, two days later, put in contact with naïve (grey) and vaccinated (red). (a) Rectal temperature was measured daily after MERS-CoV. Each line/sign represents an individual animal. One naïve (b) and one vaccinated, contact animal (c) showed moderate mucus excretion at 5-9 and 8-19 days post-inoculation procedure, respectively.

**Additional file 3.** Materials and methods.

## Data Availability

The datasets used and/or analysed during the current study are available from the corresponding author on reasonable request.

## Abbreviations

dpi: Days post inoculation
MERS-CoV: Middle East respiratory syndrome coronavirus
MPSP: Multimeric protein scaffold particles
nAbs: Neutralizing antibodies
RBD: Receptor-binding domain
S: Spike
