# Supplementary figures and images for: Protective efficacy of an RBD-based Middle East respiratory syndrome coronavirus (MERS-CoV) particle vaccine in llamas
Source: One Health Outlook. 2022 Jun 24;4:12. doi: 10.1186/s42522-022-00068-9 (PMC9225808; doi:10.1186/s42522-022-00068-9)

## Slide 1
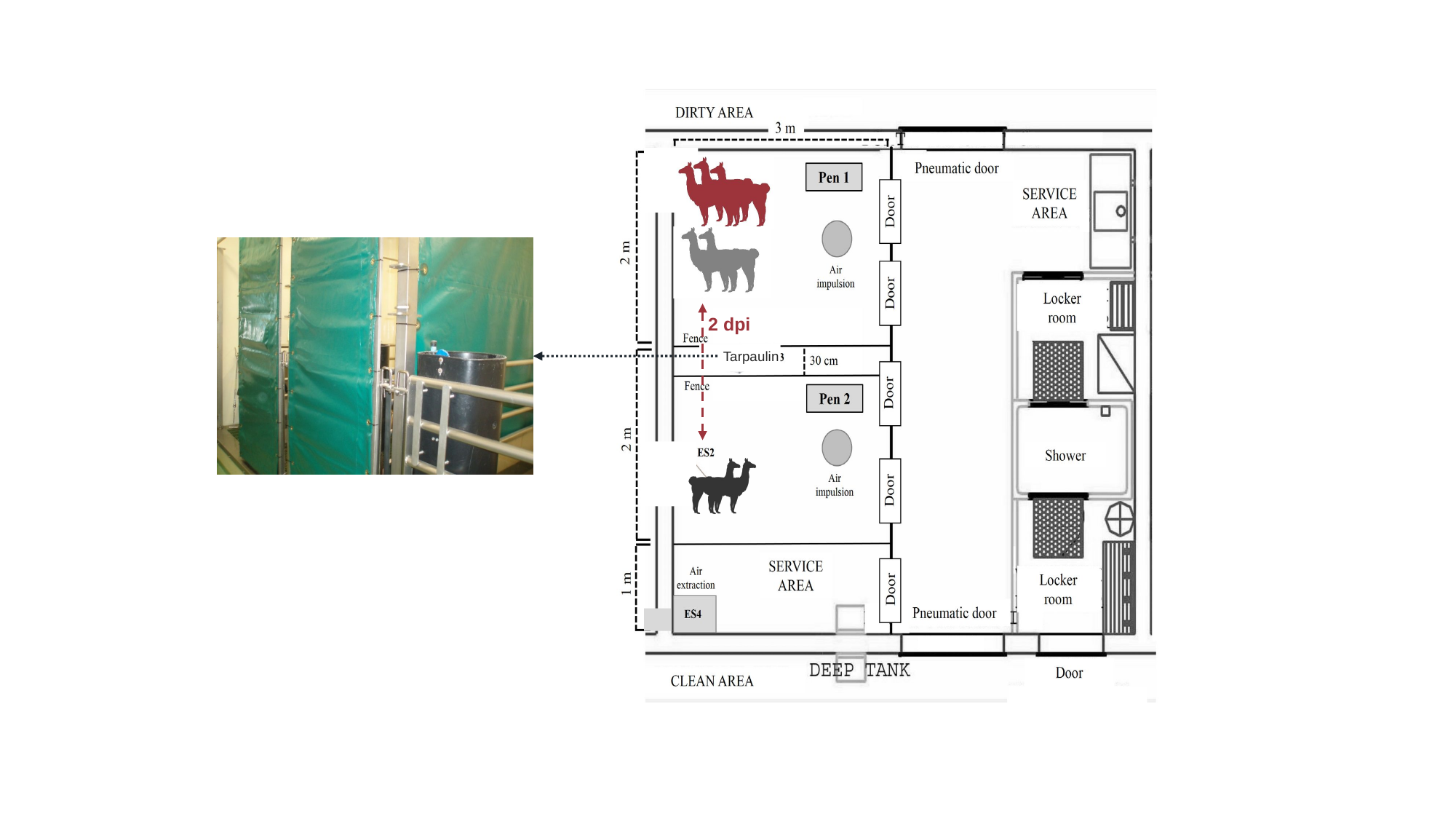

2 dpi
Tarpaulin

Supplement: Supplementary file 1 — Additional file 1: Fig. 1. Animal distribution scheme inside the experimental box. Experimental groups were kept in different compartments separated by tarpaulin to prevent animal contact. Two days after inoculation procedure, the tarpaulin was removed and experimentally infected llamas (black) were then in direct contact with naïve (grey) and vaccinated (red) animals. [file 42522_2022_68_MOESM1_ESM.pptx]

## Slide 1
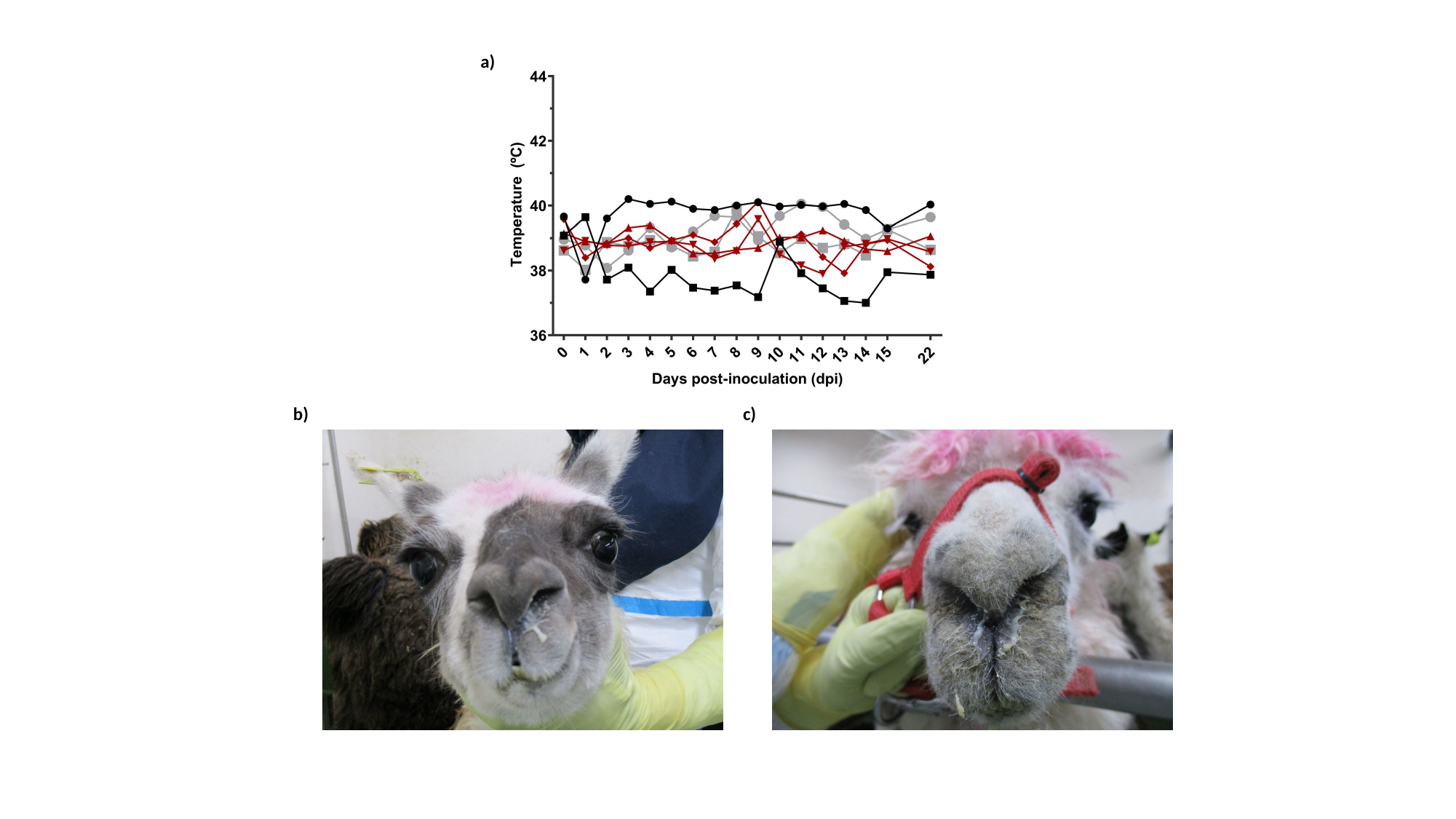

a)
b)
c)

Supplement: Supplementary file 2 — Additional file 2: Fig. 2. Temperature and rhinorrhoea after MERS-CoV exposure to llamas. MERS-CoV experimentally inoculated llamas (black) were, two days later, put in contact with naïve (grey) and vaccinated (red). (a) Rectal temperature was measured daily after MERS-CoV. Each line/sign represents an individual animal. One naïve (b) and one vaccinated, contact animal (c) showed moderate mucus excretion at 5-9 and 8-19 days post-inoculation procedure, respectively. [file 42522_2022_68_MOESM2_ESM.pptx]
