# Supplementary material for: Protective efficacy of an RBD-based Middle East respiratory syndrome coronavirus (MERS-CoV) particle vaccine in llamas
Source: One Health Outlook. 2022 Jun 24;4:12. doi: 10.1186/s42522-022-00068-9 (PMC9225808; doi:10.1186/s42522-022-00068-9)
Supplement: Supplementary file 3 — Additional file 3. Materials and methods. [file 42522_2022_68_MOESM3_ESM.doc]

**Protective efficacy of an RBD-based Middle East respiratory syndrome coronavirus (MERS-CoV) particle vaccine in llamas**

Authors: Jordi Rodon^1^, Anna Z. Mykytyn^2^, Guillermo Cantero^1^, Irina C. Albulescu^3^, Berend-Jan Bosch^3^, Alexander Brix^4^, Jean-Christophe Audonnet^5^, Albert Bensaid^1^, Júlia Vergara-Alert^1^, Bart L. Haagmans^2,*^, Joaquim Segalés^6,7,*^

Author affiliations: ^1^IRTA, Centre de Recerca en Sanitat Animal (CReSA, IRTA-UAB), Campus de la UAB, 08193 Bellaterra (Cerdanyola del Vallès), Spain; ^2^Department of Viroscience, Erasmus Medical Centre, 3000 CA Rotterdam, The Netherlands; ^3^Virology Division, Department of Infectious Diseases & Immunology, Faculty of Veterinary Medicine, Utrecht University, 3584 CL Utrecht, The Netherlands; ^4^Boehringer Ingelheim Veterinary Research Center GmbH & Co. KG, Hanover, Germany; ^5^Boehringer Ingelheim Animal Health, Global Innovation, 813 Cours du 3ème millénaire, 69380 Saint-Priest, France; ^6^UAB, CReSA (IRTA-UAB), Campus de la UAB, 08193 Bellaterra (Cerdanyola del Vallès), Spain; ^7^Departament de Sanitat i Anatomia Animals, Facultat de Veterinaria, UAB, 08193 Bellaterra (Cerdanyola del Vallès), Spain

*Correspondence and requests for materials should be addressed to B.L.H. (b.haagmans@erasmusmc.nl) and/or J.S. (joaquim.segales@irta.cat)

**Additional Materials and Methods**

**Animal welfare and ethics**

Animal experiments with MERS-CoV were performed at the biosafety level-3 (BSL-3) facilities of the Biocontainment Unit of IRTA-CReSA (Barcelona, Spain). The present study was approved by the Ethical and Animal Welfare Committee of IRTA (CEEA-IRTA) and by the Ethical Commission of Animal Experimentation of the Autonomous Government of Catalonia (file No. CEA-OH/10942/1).

**Cell culture and virus**

Vero E6 cells (CRL-1586, ATCC, USA) were cultured in Dulbecco’s modified Eagle medium (DMEM; Lonza, Switzerland) supplemented with 5% fetal calf serum (EuroClone, Italy), 100 U/mL penicillin, 100 µg/mL streptomycin, and 2 mM glutamine (all ThermoFisher Scientific, USA). A passage 3 MERS-CoV Qatar15/2015 strain stock was propagated in Vero E6 cells for 3 days at 37ºC and 5% CO_2_. The infectious virus titer was determined in Vero E6 cells and calculated by determining the dilution that caused cytopathic effect (CPE) in 50% of the inoculated cell cultures (50% tissue culture infectious dose endpoint, TCID_50_).

**Vaccine design and expression**

The vaccine immunogen was prepared by coupling purified RBD of MERS-CoV spike onto the surface of the mi3 60-mer multimeric protein scaffold particle (MPSP) using the SpyTag-SpyCatcher strategy (1,2). Recombinant mi3 fused to the SpyCatcher was expressed in *E. coli* cells, as follows. A bacterial culture with an OD_600_ of ~0.5 was induced for expression with 0.1 mM IPTG (isopropyl β-D-1-thiogalactopyranoside) and incubated overnight at 18°C in a shaking incubator. Next the bacteria were pelleted by centrifugation at 8,000 x g, incubated for 30 min in lysis buffer at 25°C, followed by sonication on ice. Unlysed bacteria, debris and the insoluble protein fraction were removed by centrifugation (100min/4°C/18,000 x g). Purification was performed by an initial heat treatment step (30 min, 60°C), followed by another centrifugation step (see above) and size exclusion chromatography (Superdex^TM^ 75). Recombinant RBD of MERS-CoV spike with a C-terminal SpyTag was expressed and purified as previously described (2) and coupled to the SpyCatcher containing mi3 MPSP at a molar ratio of 1:3 RBD:mi3, in DPBS without calcium and magnesium (Lonza). Concentrations of all purified proteins were determined with the NanoDrop ND-1000 spectrophotometer.

**Study design**

Seven healthy llamas were purchased from a private animal facility and housed at the IRTA farm of Alcarràs (Catalonia, Spain) during the immunization period. Animals were transferred to the BSL-3 animal facilities of the Biocontainment Unit of IRTA-CReSA, in Barcelona (Spain), for experimental procedures involving MERS-CoV.

Three llamas were intramuscularly immunized in the right side of the neck with 40 µg of a MERS-CoV RBD coupled with 120 µg mi3 and emulsified (1:1 volume) with Montanide™ ISA 206 VG (Seppic, France) adjuvant, administering a total volume of 2 mL per animal and dose. A second immunization was conducted 3 weeks later as described above but in the left side of the neck. Two other animals received an emulsion of PBS and Montanide™ ISA 206 VG (1:1 volume) at the vaccination days, while the two remaining animals were kept naïve. Five weeks after the first immunization, two naïve llamas were intranasally inoculated with a 10^7^ TCID_50_ of MERS-CoV Qatar15/2015 strain (GenBank Accesion MK280984) in 3 mL saline solution using a nebulization device (LMA® MADgic®, Teleflex Inc., USA), administrating 1.5 mL into each nostril. At 2 days post-inoculation (dpi) vaccinated (n=3) and naïve llamas (n=2) were brought into contact with inoculated llamas (**Fig. 1**, **Additional Fig. 1**). The box in the BSL-3 facility was set up as in previous MERS-CoV transmission studies performed in pigs and llamas (3,4).

Animals were monitored daily for respiratory clinical signs, including sneezing, coughing, nasal discharge and/or dyspnea. Rectal temperatures were recorded with a fast display digital thermometer (AccuVet®, Infratec, Italy) until 15 dpi plus the day of necropsy. Nasal swabs were obtained daily until 15 dpi, and then at 17 and 22 dpi. Whole blood samples of all animals were collected from the jugular vein using Vacutainer® tubes (Beckton Dickinson, USA) and serum samples were obtained before the first and the second immunizations, prior to challenge, and weekly after the MERS-CoV challenge. Animals were euthanized at 22 dpi with an overdose of pentobarbital and a complete necropsy was performed, with special emphasis on upper and lower respiratory tract lesions (4).

**MERS-CoV genomic and subgenomic RNA detection**

Viral RNA was extracted from nasal swab samples with the IndiMag pathogen kit (Indical Biosciences, Germany) using a Biosprint 96 workstation (Qiagen, Germany), according to the manufacturer’s instructions. The genomic and subgenomic RNA extracts were tested by the UpE and M mRNA RT-qPCR assays, respectively (5,6). Viral RNA was detected from nasal swab samples by RT-qPCR as previously described (3,4,7,8). RT-qPCR was carried out using AgPath-ID^TM^ One-Step RT-PCR Reagents (Applied Biosystems, Life Technologies, USA), and amplification was done by using a 7500 Fast Real-Time PCR System (Applied Biosystems, Life Technologies, USA) programmed as follows: 10 min at 50ºC, 10 sec at 95ºC, and 45 cycles of 15 s at 95ºC and 30 sec at 58ºC. Samples with a quantification cycle (Cq) value ≤40 were considered positive for MERS-CoV genomic or subgenomic RNA.

**Virus titration**

Nasal swabs samples with lower Cq value ≤ 30 to MERS-CoV RNA, as determined by RT-qPCR, were evaluated for the presence of infectious virus by titration in Vero cells, as previously reported (4,7–9). Ten-fold dilutions of the samples, starting with a 1/10 dilution, were transferred to Vero E6 monolayers. Plates were monitored daily under a light microscope and wells were evaluated for the presence of virus-induced cytopathic effect for 6 days. The infectious virus in nasal swabs was calculated by determining the TCID_50_/ml.

**Plaque reduction neutralization assay**

Serum samples collected at different time-points were tested for the presence of neutralizing antibodies against MERS-CoV (EMC/2012 isolate; NCBI accession number: NC_019843.3) using a plaque reduction neutralization (PRNT) assay. PRNT was performed according to a previously published protocol (4,9), with minor modifications. Briefly, samples were first inactivated at 56ºC for 30 min. Then, 50 μl of serially-diluted serum in Opti-MEM I (IX) + GlutaMAX (Gibco, USA) were mixed 1:1 with virus (400 PFU) and incubated at 37ºC for 1 hour before layering over fully confluent monolayers of Calu-3 cells (washed once prior with Opti-MEM I (IX) + GlutaMAX). After 8 h of incubation, the cells were fixed with formalin, permeabilized with 70% ethanol, washed in PBS and stained using mouse anti-MERS-CoV nucleocapsid (SinoBiological, China; diluted 1:1,000 in 0.1% bovine serum albumin (BSA) in PBS) followed by goat anti-mouse Alexa Fluor 488 antibody (Invitrogen, 1:2,000 in 0.1% BSA in PBS). Plates were scanned on the Amersham Typhoon Biomolecular Imager (GE Healthcare, USA). Data was analysed using ImageQuantTL 8.2 image analysis software (GE Healthcare). The PRNT titre was calculated using GraphPad Prism 9, calculating a 50% reduction in infected cell counts based on non-linear regression with bottom constraints of 0% and top constraints of 100%.

**References**

1. Bruun TUJ, Andersson A-MC, Draper SJ, Howarth M. Engineering a Rugged Nanoscaffold To Enhance Plug-and-Display Vaccination. ACS Nano. 2018 Sep 25;12(9):8855–66. Available from: https://doi.org/10.1021/acsnano.8b02805

2. Okba NMA, Widjaja I, van Dieren B, Aebischer A, van Amerongen G, de Waal L, et al. Particulate multivalent presentation of the receptor binding domain induces protective immune responses against MERS-CoV. Emerg Microbes Infect. 2020 Jan 1;9(1):1080–91. Available from: https://doi.org/10.1080/22221751.2020.1760735

3. Vergara-Alert J, Raj VS, Muñoz M, Abad FX, Cordón I, Haagmans BL, et al. Middle East respiratory syndrome coronavirus experimental transmission using a pig model. Transbound Emerg Dis. 2017 Oct 1;64(5):1342–5. Available from: https://doi.org/10.1111/tbed.12668

4. Rodon J, Okba NMA, Te N, van Dieren B, Bosch B-J, Bensaid A, et al. Blocking transmission of Middle East respiratory syndrome coronavirus (MERS-CoV) in llamas by vaccination with a recombinant spike protein. Emerg Microbes Infect. 2019 Jan 1;8(1):1593–603. Available from: https://doi.org/10.1080/22221751.2019.1685912

5. Corman VM, Eckerle I, Bleicker T, Zaki A, Landt O, Eschbach-Bludau M, et al. Detection of a novel human coronavirus by real-time reverse-transcription polymerase chain reaction. Euro Surveill. 2012;

6. Coleman CM, Frieman MB. Growth and Quantification of MERS-CoV Infection. Curr Protoc Microbiol. 2015 Nov 1;37(1):15E.2.1-15E.2.9. Available from: https://doi.org/10.1002/9780471729259.mc15e02s37

7. Vergara-Alert J, van den Brand JMA, Widagdo W, Muñoz M, Raj S, Schipper D, et al. Livestock Susceptibility to Infection with Middle East Respiratory Syndrome Coronavirus. Emerg Infect Dis J. 2017;23(2):232. Available from: https://wwwnc.cdc.gov/eid/article/23/2/16-1239_article

8. Te N, Rodon J, Ballester M, Pérez M, Pailler-García L, Segalés J, et al. Type I and III IFNs produced by the nasal epithelia and dimmed inflammation are features of alpacas resolving MERS-CoV infection. PLOS Pathog. 2021 May 24;17(5):e1009229. Available from: https://doi.org/10.1371/journal.ppat.1009229

9. Haagmans BL, van den Brand JMA, Raj VS, Volz A, Wohlsein P, Smits SL, et al. An orthopoxvirus-based vaccine reduces virus excretion after MERS-CoV infection in dromedary camels. Science. 2016;351(6268):77–81. Available from: http://www.sciencemag.org/cgi/doi/10.1126/science.aad1283
